# The association of serum neuron-specific enolase with other disease markers in chronic obstructive pulmonary disease: A case-control study

**DOI:** 10.12669/pjms.345.15145

**Published:** 2018

**Authors:** Jie Li, Xinyi Kong, Wei Shu, Wei Zhang

**Affiliations:** 1 Jie Li, MD. Medical School of Nanchang University, Nanchang, China, Department of Internal Medicine, Jiangxi Provincial Chest Hospital; 2 Xinyi Kong, MSc. Department of Internal Medicine, The Second Affiliated Hospital of Nanchang University, China; 3 Wei Shu, MSc. Department of Internal medicine, Jiangxi Provincial Chest Hospital, Nanchang, China; 4 Wei Zhang, MD. Department of Respiration, The First Affiliated Hospital of Nanchang University, China

**Keywords:** Association, Serum, Neuron-specific enolase, Chronic obstructive pulmonary disease

## Abstract

**Objective:**

The aim of the present study was to investigate the association between serum neuron-specific enolase (sNSE) levels and gender, age, body mass index (BMI) in patients with chronic obstructive pulmonary disease (COPD).

**Methods:**

This case-control study was carried out among 182 participants in Jiangxi Provincial chest hospital, Nanchang, China, in 2017. One hundred and two patients diagnosed with COPD based on the Global Initiative for Chronic Obstructive Lung Disease (GOLD) grading classification and 80 Non-COPD participants were recruited. Multivariate logistic regression analysis was employed to examine whether or not sNSE and other indicators were independently associated with COPD.

**Results:**

Serum NSE levels were not significantly different between the two groups (P=0.08). Whereas in COPD sub-groups, the levels of sNSE increased parallelly in a GOLD stage-dependent manner. There was a positive correlation between PH, P_O2_, pack-years, FEV_1_ and the presence of COPD, but there was no significant correlation between sNSE, P_CO2_ and the presence of COPD.

**Conclusions:**

Serum NSE gradually increased with the severity of COPD and its change reflected changes in brain cells. PH, P_O2_, pack-years and forced expiratory volume in one second (FEV1), were independent risk factors for COPD patients.

## INTRODUCTION

Chronic obstructive pulmonary disease (COPD) is a leading cause of global morbidity and mortality resulting in social and economic burdens.^1^,^2^ It is characterized by persistent airflow obstruction due to airway and /or alveolar abnormalities and can easily progress to respiratory failure manifested as hypoxemia and carbondioxide retention. Brain cells are extremely sensitive to hypoxia and carbondioxide (CO_2_) retention, which can lead to acidosis and then central inhibition of brain cells, furthermore causes damages to brain tissue and nerve cells, resulting in brain cell dysfunction and metabolic disorders. The current diagnosis of respiratory failure depended mainly on blood gas analysis, but the assessment of the degree of nervous system damage mainly depended on the subjective assessment of clinicians, which lacked objective indicators. If some indicator changes can be observed in the early stages of respiratory failure, then timely intervention will have more important clinical significance for reducing or avoiding the progression to hypercapnic encephalopathy syndrome.

Neuron-specific enolase (NSE) is one of the enolase enzymes involved in the glycolytic pathway, found in nerve tissues and neuroendocrine tissues.^3^ NSE has the highest activity in brain tissue cells and is one of the specific markers reflecting the damage of the nervous system, after the damage of the central nervous system. NSE can be released into the blood, and cerebrospinal fluid through the damaged cell membrane and blood-brain barrier.^4^ Evidence has shown bronchial epithelial cells, and type II pneumocytes contain NSE,^5^ which could be successfully employed as a marker to identify small cell lung cancer.^6^ Recently NSE was considered as a good candidate in benign pulmonary disease.^7^ Barouchos compared the performance of Serum NSE (sNSE) in COPD exacerbation patients (severity C and D), and found that sNSE was closely related to some inflammatory biomarkers such as erythrocyte sedimentation rate, C-reactive protein, as well as white blood cells count.^7^ However, the relationship between sNSE and all COPD classification patients, particularly those with mild symptoms, and some clinical observation index (FEV_1_, PH, P_O2_, P_CO2_, etc.) was still not clear.

Therefore, this study aims to investigate the changes of sNSE at different stages of COPD, compared with the corresponding changes in patients with some observed indicators, to find the relationship between these indicators and COPD, to assess the severity of brain injury in patients with COPD, so as to provide a clinical reference for diagnosis and treatment.

## METHODS

A case-control study was carried out among 182 participants in Jiangxi Provincial chest hospital, Nanchang, China, in 2017. Their personal information such as gender, age, body weight, Body Mass Index (BMI), sNSE, pack-years, forced expiratory volume in 1second (FEV1), the potential of hydrogen (pH), P_O2_, P_CO2_ were collected. One hundred and two were diagnosed with COPD, and they were further classified into stage A to D based on 2017 Global Initiative for Chronic Obstructive Lung Disease (GOLD) Standard.^8^ Candidates for inclusion in the model were current smokers. Pack-years of smoking, FEV_1_, arterial blood gas analysis including pH, P_O2_ and P_CO2_ were recorded. Key exclusion criteria were: coexisting illnesses that could preclude participation in the study or interfere with the study results. The study protocol was approved by the ethics committee, and all patients provided written informed consent.

### Laboratory Measurements

Pulmonary function, sNSE, and arterial blood gas analysis in patients with COPD were obtained just before the start of treatment; the levels of sNSE were examined within 2 hours for subsequent assay.^9^

### Statistical Analysis

All data were presented as mean± standard deviation or median (25^th^ -75^th^ percentile). Analyses were done with SPSS23.0 (IBM, Armonk, NY, USA) and Graphpadprism 5.0 (Graph Pad Software, LaJolla, CA, USA). Multivariate logistic regression analysis was employed to examine whether or not sNSE was independently associated with COPD, P-values ≤ 0.05 were considered statistically significant. This trial was registered at http://www.chictr.org.cn with number ChiCTR-ROC-17013587.

## RESULTS

The characteristics of 182 participants are shown in Table-I. Age, body weight, and BMI did not show any significant difference between the two groups. sNSE levels were not importantly different between two groups (P=0.08).

**Table-I:** Clinical characteristics of participants.

	All subjects	COPD group	Non-COPD group	P- value
Patients (n)	182	102	80	–
Male	65.9%	76.5%	52.5%	0.001
Age(years)	63.42±12.88	62.34±13.61	64.80±11.83	0.20
Bodyweight	53.23±9.01	56.02±90.09	54.21±7.82	0.18
BMI	20.28±2.34	20.29±2.49	20.27±2.13	0.96
NSE	17.25±4.20	17.94±4.57	16.35±3.49	0.08

Values were expressed as mean±standard deviation or median (25^th^ - 75^th^ percentile)

Various clinical parameters in patients with COPD of different stages are shown in Table-II. The smallest group was formed by patients in stage A. GOLD D contained the highest proportion of current smokers and pack-years. The number of different types of FEV_1_, pH, and P_O2_ decreased from stage A to D. P_CO2_ levels in these four groups did not reach statistical significance (P=0.13). Levels of sNSE increased parallelly from stage A to D.

**Table-II:** Clinical parameters in COPD group patients with different stages.

	GOLD A	GOLD B	GOLD C	GOLD D	P-value

	(n=14)	(n=27)	(n=32)	(n=29)	
Age	66.86	69.07	60.81	55.59	0.001
Male	85.7%	77.8%	75%	72.4%	<0.001
BMI	20.46	20.12	20.26	20.40	0.97
Current smoker	33.3%	38.1%	30%	40%	0.001
Pack-years	35.74	39.21	37.22	41.69	<0.001
FEV_1_ (as a % of predicted)	72.03	58.63	51.59	35.29	<0.001
pH	7.42	7.39	7.36	7.35	<0.001
P_O2_	74	66.81	64.09	42.07	<0.001
P_CO2_	47.71	52.19	60.28	55.41	0.13
sNSE	13.25	16.46	19.22	20.17	<0.001

Then the possible associations between sNSE and other factors were investigated using multivariate logistic regression analysis as shown in Table-III, which included age, body weight, BMI, pH, P_O2_, P_CO2_, current smokers, pack-years, FEV_1_ and sNSE as independent variables, showed that pH, P_O2_, P_CO2_, current smokers, pack-years and FEV_1_ emerged as significant and independent factors associated with the presence of COPD.

**Table-III:** Multiple logistic regression analysis of factors associated with COPD.

	OR	95%CI	P-value
Age (years)	0.95	0.89-1.01	0.08
Body weight (kg)	1.05	0.88-1.25	0.61
BMI	0.74	0.39-1.39	0.35
pH	2.00 E+10	4367.09-9.15E+16	0.002
P_O2_	0.92	0.88-0.97	0.003
P_CO2_	1.07	1.02-1.13	0.01
Current smoker	0.17	0.04-0.74	0.02
Pack-years	1.30	1.14-1.47	<0.001
FEV_1_(as a% of predicted)	0.93	0.89-0.97	<0.001
sNSE(ng/ml)	1.09	1.01-1.18	0.08

OR: odds ratio; 95% CI: 95% confidence intervals.

## DISCUSSION

NSE has been proved to exist in bronchial epithelial cells and type II pneumocytes,^5^ which can be used as a distinguishing marker to identify small cell lung cancer and non-small cell lung cancer.^6^ Recent research^7^ has demonstrated NSE was also elevated in patients with benign pulmonary diseases. We found that sNSE, even though statistically significant in patients with COPD, did not exceed the normal range of sNSE (0-20ng/ml), suggesting that there may be some regulatory mechanism that inhibits further release of sNSE.

Speaking of pack-years, the present study showed a positive correlation between pack-years and sNSE. The reason may be attributed to that, on the one hand, smoking can affect the neurotransmitter function and the balance of oxidative stress in vivo.^10^ On the other hand, smoking increases the level of synaptic dopamine, a vital source of central free radical.^11^ However, our data did not show a positive correlation between sNSE and current smoking. This indirectly showed that for COPD patients with a long-time smoking history, the effects of tobacco on the cranial nerves did not disappear immediately after quitting smoking.

Regarding P_O2_, P_CO2,_ and pH, abnormal brain state was associated with hypoxia, hypercapnia, and acidosis as expected. Among the three factors, P_O2_ and pH were more closely related to sNSE, which was similar to the study on perinatal newborns.^12^ Previous studies had shown that hypercapnia can cause pulmonary encephalopathy and even coma, but Bain^13^ demonstrated in his study that hypoxemia could play a more important role in the cerebral free-radical formation and the corresponding implications for brain structure and function. Therefore, our research further confirmed hypoxia had a stronger influence on the cranial nerves compared with hypercapnia.

With regard to FEV_1_, it was significantly positively correlated with COPD, and the differences were statistically significant between COPD stages. However, no significant differences existed between stage B and stage C. The reason may be due to mMRC score,^14^ and COPD Assessment Test^15^ were included as new assessment tools in 2017 GOLD standard compared with the previous COPD guidelines, and FEV_1_ itself lacked sufficient precision as a clinical predictor to fully reflect the severity of the symptoms of COPD patients.^16^,^17^ Although a growing number of large clinical studies^18^-^20^ have shown the instability and limitations of the COPD classification, our study suggested that it remain a reference COPD classification tool. More importantly, it remained irreplaceable as a gold standard in the diagnosis of COPD.

With regard to gender, age, body weight and BMI, previous studies showed sNSE levels in cerebrospinal fluid and age were positively associated.^21^,^22^ Furtherly, Hoffmann found the elder female revealed a significant association with sNSE.^23^ However, no similar evidence was found in our study, the reason may be that the majority of COPD patients were middle-aged and elderly male patients, and the population included in the present study was not statistically significant. Some scholars even believed that obesity (BMI<25) and sNSE have a positive correlation because fat can cause damage to the brain’s grey matter density.^24^ Apparently, we did not find the same trend in the present study, and the reason may be that most elderly people have a lighter weight and long-term smoking also leads to weight loss.^25^

### Limitations of the study

First, the exclusion of any malignancy of participants was based on medical history and conventional chest and brain CT with no generalized examination, especially histological examination. Therefore, it cannot be excluded that some potential tumors affected the level of sNSE. Second, there was bias in terms of age and gender statistics, as most people with COPD in China are middle-aged and senior men. Lastly, all recruits came from Jiangxi province of China and sample size was relatively small. Therefore, multicenter studies with a larger sample size will be required in the future.

## CONCLUSIONS

Serum NSE gradually increased with the severity of COPD and its change reflected changes in brain cells pH, P_O2_, pack-years, and FEV_1_ were independent risk factors for COPD patients.

### Authors’ contributions

**JL:** Recruited the patients, designed, data collection, collected samples, and manuscript writing.

**XK:** Designed the study.

**WS:** Performed laboratory-based assays.

**WZ:** Designed the study, analyzed the data, and manuscript writing.

All authors read and approved the final manuscript.

## References

[1] Lin YH, Wang WY, Hu SX, Shi YH (2016). Serum C-reactive protein level in COPD patients stratified according to GOLD 2011 grading classification. Pak J Med Sci.

[2] Almagro P, Soriano JB, Cabrera FJ, Boixeda R, Alonso-Ortiz MB, Barreiro B (2014). Short- and medium-term prognosis in patients hospitalized for COPD exacerbation:the CODEX index. Chest.

[3] Isgro MA, Bottoni P, Scatena R (2015). Neuron-Specific Enolase as a Biomarker:Biochemical and Clinical Aspects. Adv Exp Med Biol.

[4] Nan C, Guo L, Zhao Z, Ma S, Liu J, Yan D (2016). Tetramethylpyrazine induces differentiation of human umbilical cord-derived mesenchymal stem cells into neuron-like cells in vitro. Int J Oncol.

[5] Slodkowska J, Szturmowicz M, Giedronowicz D, Rudzinski P, Sakowicz A, Kupis W (1997). Trophoblastic markers and CEA in non-small cell lung cancer:the comparison studies of tumour cells expression and serum concentration. Rocz Akad Med Bialymst.

[6] Wang R, Wang G, Zhang N, Li X, Liu Y (2013). Clinical evaluation and cost-effectiveness analysis of serum tumor markers in lung cancer. Biomed Res Int.

[7] Barouchos N, Papazafiropoulou A, Iacovidou N, Vrachnis N, Armeniakou E, Dionyssopoulou V (2015). Comparison of tumor markers and inflammatory biomarkers in chronic obstructive pulmonary disease (COPD) exacerbations. Scand J Clin Lab Invest.

[8] (2017). Global strategy for the diagnosis, management, and prevention of chronic obstructive pulmonary disease (2017 Report).

[9] Azabou E, Magalhaes E, Braconnier A, Yahiaoui L, Moneger G, Heming N (2015). Early Standard Electroencephalogram Abnormalities Predict Mortality in Septic Intensive Care Unit Patients. PLoS One.

[10] Isik B, Ceylan A, Isik R (2007). Oxidative stress in smokers and non-smokers. Inhal Toxicol.

[11] Brody AL, Olmstead RE, London ED, Farahi J, Meyer JH, Grossman P (2004). Smoking-induced ventral striatum dopamine release. Am J Psychiatry.

[12] Verdu Perez A, Falero MP, Arroyos A, Estevez F, Felix V, Lopez Y (2001). Blood neuronal specific enolase in newborns with perinatal asphyxia]. Rev Neurol.

[13] Bain AR, Ainslie PN, Hoiland RL, Barak OF, Drvis I, Stembridge M (2017). Competitive apnea and its effect on the human brain:focus on the redox-regulation of blood-brain barrier permeability and neuronal-parenchymal integrity. FASEB J.

[14] CM F (1960). Standardised questionnaire on respiratory symptoms:a statement prepared and approved by the MRC Committee on the Aetiology of Chronic Bronchitis (MRC breathlessness score). BMJ.

[15] Jones PW, Harding G, Berry P, Wiklund I, Chen WH, Kline Leidy N (2009). Development and first validation of the COPD Assessment Test. Eur Respir J.

[16] Decramer M, Celli B, Kesten S, Lystig T, Mehra S, Tashkin DP (2009). Effect of tiotropium on outcomes in patients with moderate chronic obstructive pulmonary disease (UPLIFT):a prespecified subgroup analysis of a randomised controlled trial. Lancet.

[17] Hurst JR, Vestbo J, Anzueto A, Locantore N, Mullerova H, Tal-Singer R (2010). Susceptibility to exacerbation in chronic obstructive pulmonary disease. N Engl J Med.

[18] Kim J, Yoon HI, Oh YM, Lim SY, Lee JH, Kim TH (2015). Lung function decline rates according to GOLD group in patients with chronic obstructive pulmonary disease. Int J Chron Obstruct Pulmon Dis.

[19] Soriano JB, Lamprecht B, Ramirez AS, Martinez-Camblor P, Kaiser B, Alfageme I (2015). Mortality prediction in chronic obstructive pulmonary disease comparing the GOLD 2007 and 2011 staging systems:a pooled analysis of individual patient data. Lancet Respir Med.

[20] Aaron SD, Tan WC, Bourbeau J, Sin DD, Loves RH, MacNeil J (2017). Diagnostic Instability and Reversals of Chronic Obstructive Pulmonary Disease Diagnosis in Individuals with Mild to Moderate Airflow Obstruction. Am J Respir Crit Care Med.

[21] Streitburger DP, Arelin K, Kratzsch J, Thiery J, Steiner J, Villringer A (2012). Validating serum S100B and neuron-specific enolase as biomarkers for the human brain - a combined serum, gene expression and MRI study. PLoS One.

[22] Hajdukova L, Sobek O, Prchalova D, Bilkova Z, Koudelkova M, Lukaskova J (2015). Biomarkers of Brain Damage:S100B and NSE Concentrations in Cerebrospinal Fluid--A Normative Study. Biomed Res Int.

[23] Hoffmann J, Janowitz D, Van der Auwera S, Wittfeld K, Nauck M, Friedrich N (2017). Association between serum neuron-specific enolase, age, overweight, and structural MRI patterns in 901 subjects. Transl Psychiatry.

[24] Mueller K, Sacher J, Arelin K, Holiga S, Kratzsch J, Villringer A (2012). Overweight and obesity are associated with neuronal injury in the human cerebellum and hippocampus in young adults:a combined MRI, serum marker and gene expression study. Transl Psychiatry.

[25] Chen H, Hansen MJ, Jones JE, Vlahos R, Bozinovski S, Anderson GP (2006). Cigarette smoke exposure reprograms the hypothalamic neuropeptide Y axis to promote weight loss. Am J Respir Crit Care Med.

